# Analysis of progression-free survival of first-line tyrosine kinase inhibitors in patients with non-small cell lung cancer harboring leu858Arg or exon 19 deletions

**DOI:** 10.18632/oncotarget.13815

**Published:** 2016-12-07

**Authors:** Feng-Che Kuan, Shih-Hong Li, Chih-Liang Wang, Meng-Hung Lin, Ying-Huang Tsai, Cheng-Ta Yang

**Affiliations:** ^1^ Department of Hematology and Oncology, Department of Medicine, Chang-Gung Memorial Hospital, Chiayi 61363, Taiwan; ^2^ Graduate Institute of Clinical Medical Sciences, Chang-Gung University, Taoyuan 33302, Taiwan; ^3^ Department of Thoracic Medicine, Chang-Gung Memorial Hospital, Taoyuan 33305, Taiwan; ^4^ Department of Medicine, Chang-Gung University, Taoyuan 33302, Taiwan; ^5^ Cencer of Excellence for Chang Gung Research Datalink, Chang-Gung Memorial Hospital, Chiayi 61363, Taiwan; ^6^ Division of Respiratory and Critical Care Medicine, Department of Medicine, Chang-Gung Memorial Hospital, Chiayi 61363, Taiwan; ^7^ Department of Respiratory Therapy, Chang-Gung University, Taoyuan 33302, Taiwan

**Keywords:** gefitinib, erlotinib, afatinib, Leu858Arg, Thr790Met

## Abstract

**Background:**

Gefitinib, erlotinib and afatinib provide remarkable response rates and progression-free survival compared to platinum-based chemotherapy in patients with non-small cell lung cancer harboring epidermal growth factor receptor-activating mutations, and are therefore standard first-line treatment in these patients. However, no study has compared these drugs regarding progression-free survival.

**Materials and Methods:**

We conducted this retrospective study at a single medical center in Taiwan from February 16, 2011 to October 30, 2015. We used the Kaplan-Meier method to estimate survival, and multivariate Cox proportional hazard models to estimate adjusted hazard ratios and 95% confidence intervals.

**Findings:**

Of the 1006 patients diagnosed with stage IIIb and IV non-small cell lung cancer in the study period, 448 (44.5%) had EGFR-activating mutations and received first-line therapy with gefitinib (*n* = 304, 67.6%), erlotinib (*n* = 63, 14.3%), or afatinib (*n* = 81, 18.1%). The median duration of follow-up for progression-free survival was 12.1 months in the gefitinib arm (Interquartile range [IQR]: 5.5–16.5), 11.2 months in the erlotinib arm (IQR: 4.9–16.7), and 10.3 months in the afatinib arm (IQR: 7.0–14.2). Progression-free survival was significantly longer in the patients who received afatinib or erlotinib compared to those who received gefitinib (log-rank test, *p* < 0.001), and the median progression-free survival was 11.4 months in the gefitinib group.

**Interpretation:**

Afatinib and erlotinib provide significant benefits in progression-free survival compared to gefitinib in first-line treatment of patients with non-small-cell lung cancers harboring EGFR-activating mutations. Further clinical trials are warranted to validate these findings.

## INTRODUCTION

Targeting epidermal growth factor receptor (EGFR) and downstream signaling transduction has been shown to be beneficial in the treatment of lung cancer, which accounts for 19.4% of all cancer-related deaths worldwide [1, 2]. Gefitinib (Iressa^®^, marketed by AstraZeneca) is the first tyrosine kinase inhibitor, which acts by binding to the adenosine triphosphate (ATP) binding site of this enzyme [3, 4]. Erlotinib (Tarceva^®^, marketed by Roche), another first-generation EGFR tyrosine kinase inhibitor, also inhibits the formation of phosphotyrosine residues and initiation of subsequent signal cascades [5]. Afatinib (Giotrif^®^, marketed by Boehringer Ingelheim), a second-generation EGFR tyrosine kinase inhibitor, unlike gefitinib and erlotinib, provides irreversible inhibition of ATP binding by forming permanent covalent bonds, and it has been shown to be active in preclinical study against mutations such as Thr790Met [6], which have been shown to contribute to primary and acquired resistance to reversible tyrosine kinase inhibitors [7–9]. De novo Thr790Met is more likely to coexist with Leu858Arg than with exon 19 deletions, and these two mutations account for around 90% of EGFR-activating mutations [10, 11]. All of these tyrosine kinase inhibitors have shown remarkable response rates and benefits in progression-free survival compared to first-line conventional platinum-based chemotherapy [12–21], and thus they have become the standard treatment for patients with metastatic non-small-cell lung cancer harboring EGFR-activating mutations [22].

A recent phase III randomized controlled trial, LUX-Lung 7, reported that afatinib had significant benefits in progression-free survival (HR, 0.73; 95% CI, 0.57–0.95; *p* = 0.017) compared to gefitinib in patients with EGFR-mutated metastatic non-small-cell lung cancer [23]. In addition, the ARCHER 1050 (ClinicalTrials.gov Identifier NCT01774721) trial comparing another irreversible tyrosine kinase inhibitor, dacomitinib to gefitinib is currently ongoing. However, phase III randomized controlled trials mainly enroll patients with a good performance, and no trial has compared these three tyrosine kinase inhibitors together. Tyrosine kinase inhibitors have been shown to provide dramatic benefits in response rates, and provide benefits to patients presenting with visceral crisis and impaired performance status in real world practice. Therefore, we conducted this retrospective study to elucidate the efficacy of these three tyrosine kinase inhibitors as first-line treatment in patients with EGFR-mutated non-small cell lung cancer.

## RESULTS

**Figure 1:**
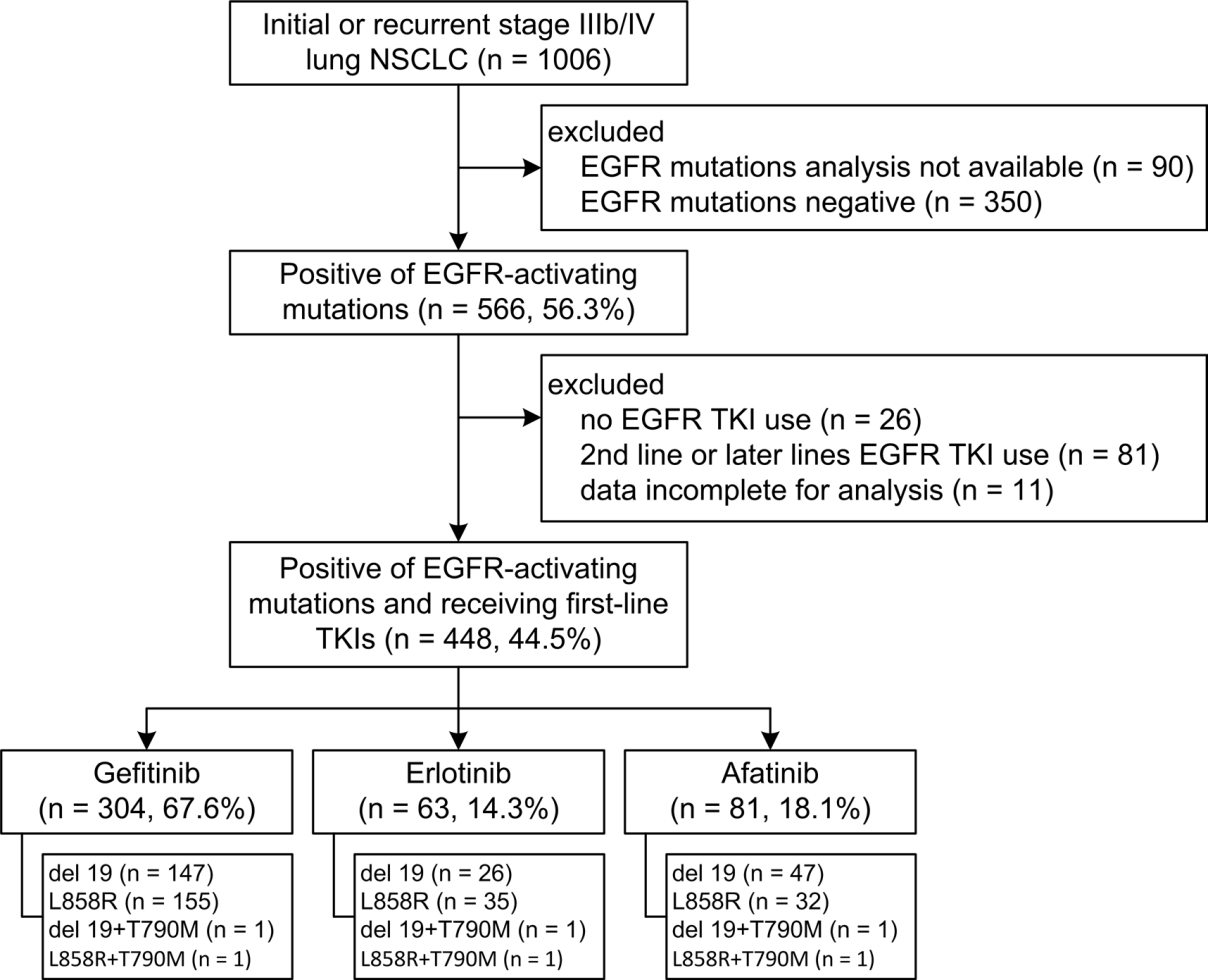
Patient disposition

**Figure 2:**
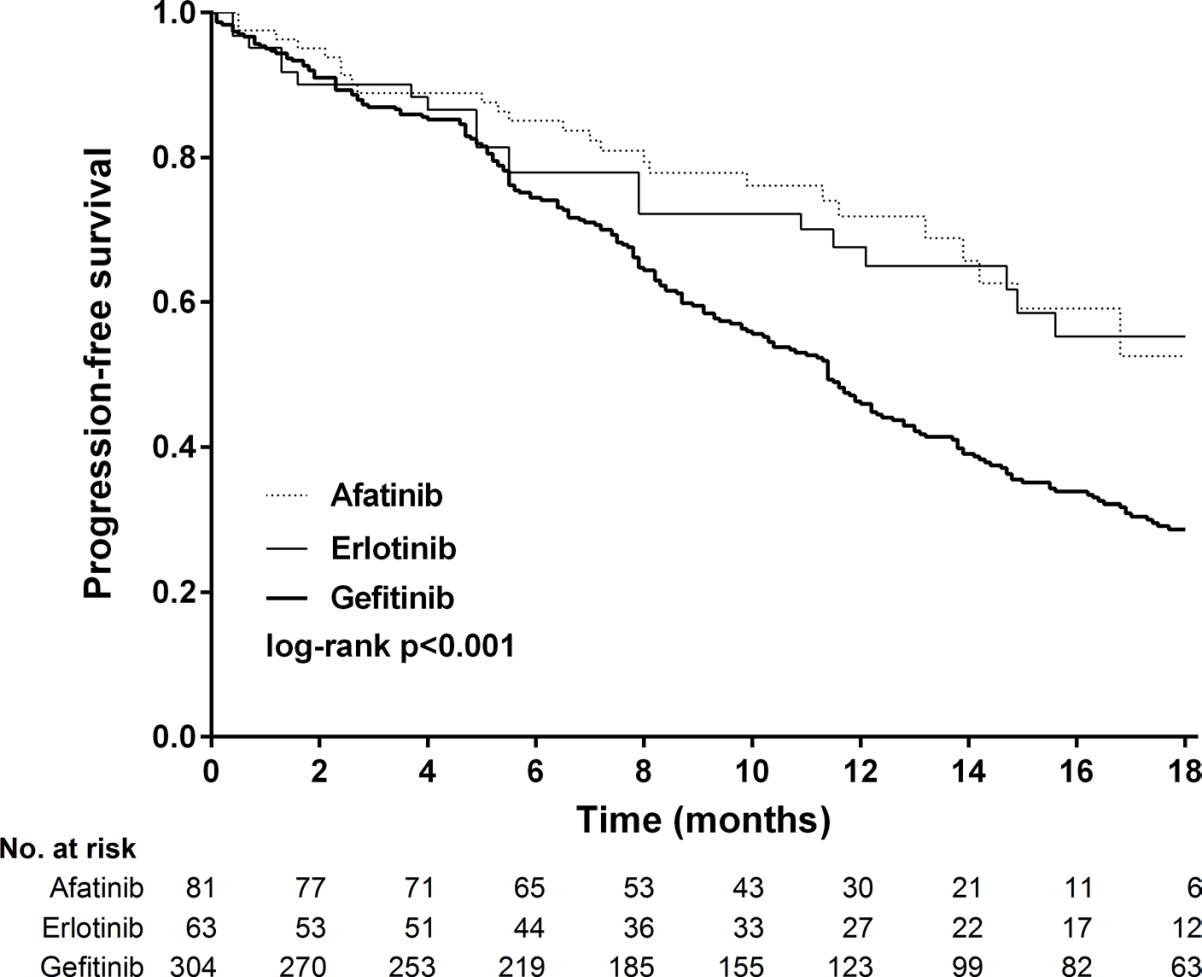
Kaplan-Meier survival curves of progression-free survival in patients received gefitinib, erlotinib and afatinib

**Figure 3:**
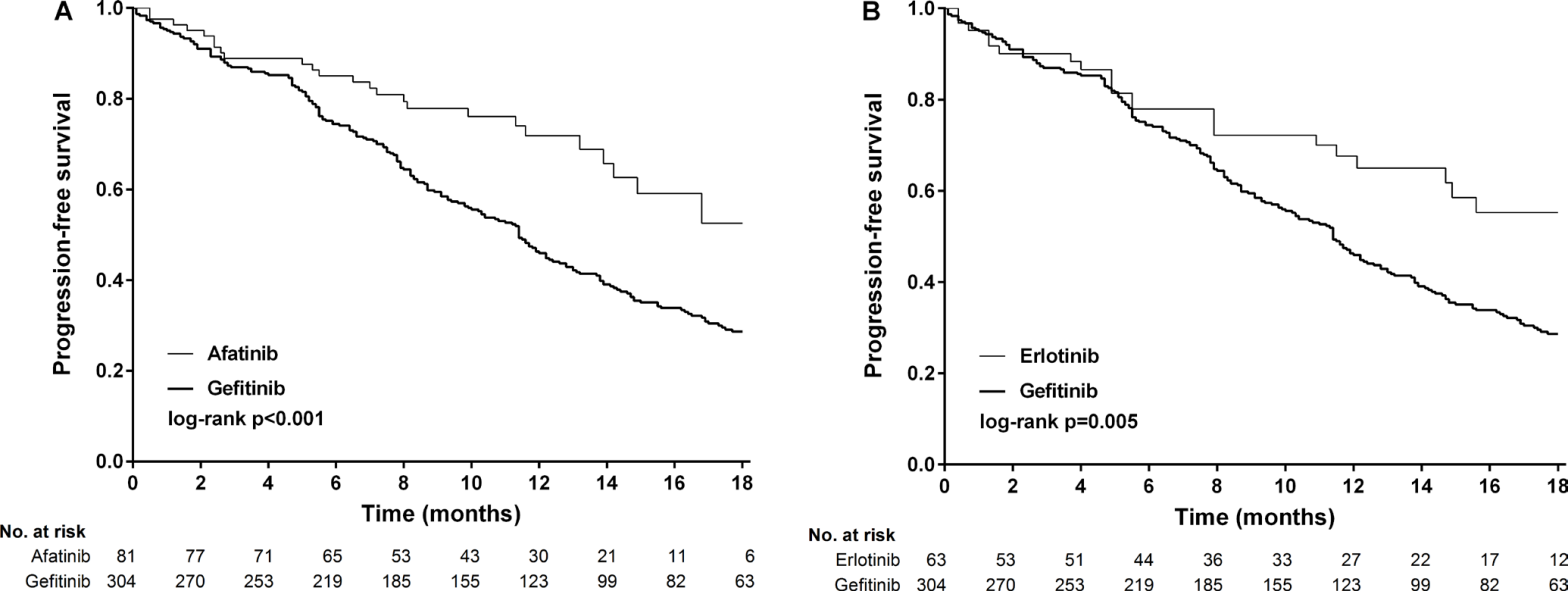
Kaplan-Meier survival curves of progression-free survival in patients received (A) afatinib and gefitinib and (B) erlotinib and gefitinib

Between February 16, 2011 and October 30, 2015, 1006 patients were screened, 448 (44.5%) of whom had newly diagnosed or recurrent stage IIIb/IV lung adenocarcinoma and received first-line gefitinib (*n* = 304), erlotinib (*n* = 63), or afatinib (*n* = 81) (Figure 1). Baseline demographics were similar between the treatment groups, except for a slight imbalance in sex (*p* = 0.213) and performance status (> 1, 24% in the gefitinib arm, *p* = 0.017, Table 1). The composite of exon 19 deletions or Leu858Arg in each arm was not statistically significant (*p* = 0.119), albeit a slightly higher percentage of exon 19 deletions (59.3%) in the afatinib group. The frequency of compound mutations were listed in Supplementary Table S2. The median outpatient dosages of gefitinib, erlotinib and afatinib were 248 mg/day (IQR, 238–250), 149 mg/day (IQR, 140–150), and 39 mg/day (IQR, 32– 40), respectively. The median durations of follow-up for progression-free survival were 12.1 months in the gefitinib arm (IQR 5.5–16.5), 11.2 months in the erlotinib arm (IQR 4.9–16.7), and 10.3 months in the afatinib arm (IQR 7.0–14.2). After 18 months of progression-free survival, 63 (20.7%) patients were still receiving treatment in the gefitinib arm, compared to 12 (19.0%) patients in the erlotinib arm and six (7.4%) in the afatinib arm. Progression-free survival was significantly longer in the patients who received afatinib or erlotinib compared to those who received gefitinib (log-rank test, *p* = 0.0001, Figure 2). The median progression-free survival was not reached in the afatinib and erlotinib groups, and 11.4 months in the gefitinib group (afatinib versus gefitinib, *p* < 0.001 and erlotinib versus gefitinib *p* = 0.005, respectively, Figure 3A and 3B).

**Table 1:** Baseline Characteristics for NSCLC by EGFR-TKIs

	EGFR-TKIs						*P*-value
	Gefitinib		Erlotinib		Afatinib		
	n	(%)	n	(%)	n	(%)	
Total	304		63		81		
Sex							0.213
Men	114	(37.5)	24	(38.1)	39	(48.1)	
Women	190	(62.5)	39	(61.9)	42	(51.9)	
Age (years)							0.095
< 65	154	(50.7)	34	(54.0)	52	(64.2)	
≥ 65	150	(49.3)	29	(46.0)	29	(35.8)	
Mean (range)	65	(33–93)	67	(47–90)	64	(37–83)	0.191
Smoking							0.802
Never	226	(74.3)	48	(76.2)	63	(77.8)	
Current or ever	78	(25.7)	15	(23.8)	18	(22.2)	
Clinical stage							0.449
IIIb	16	(5.3)	5	(7.9)	7	(8.6)	
IV	288	(94.7)	58	(92.1)	74	(91.4)	
EGFR mutation							0.119
Del19	148	(48.7)	27	(42.9)	48	(59.3)	
L858R	156	(51.3)	36	(57.1)	33	(40.7)	
Baseline brain metastases							0.867
Absence	244	(80.3)	52	(82.5)	64	(79.0)	
Presence	60	(19.7)	11	(17.5)	17	(21.0)	
ECOG PS							0.017
0 & 1	231	(76.0)	56	(88.9)	70	(86.4)	
> 1	73	(24.0)	7	(11.1)	11	(13.6)	
Grade							0.139
1	59	(19.4)	12	(19.4)	25	(30.9)	
2	64	(21.1)	19	(30.2)	21	(25.9)	
3	49	(16.1)	9	(14.3)	9	(11.1)	
missing	132	(43.4)	23	(36.5)	26	(32.1)	

Abbreviations: ECOG PS, Eastern Cooperative Oncology Group performance status.

**Figure 4:**
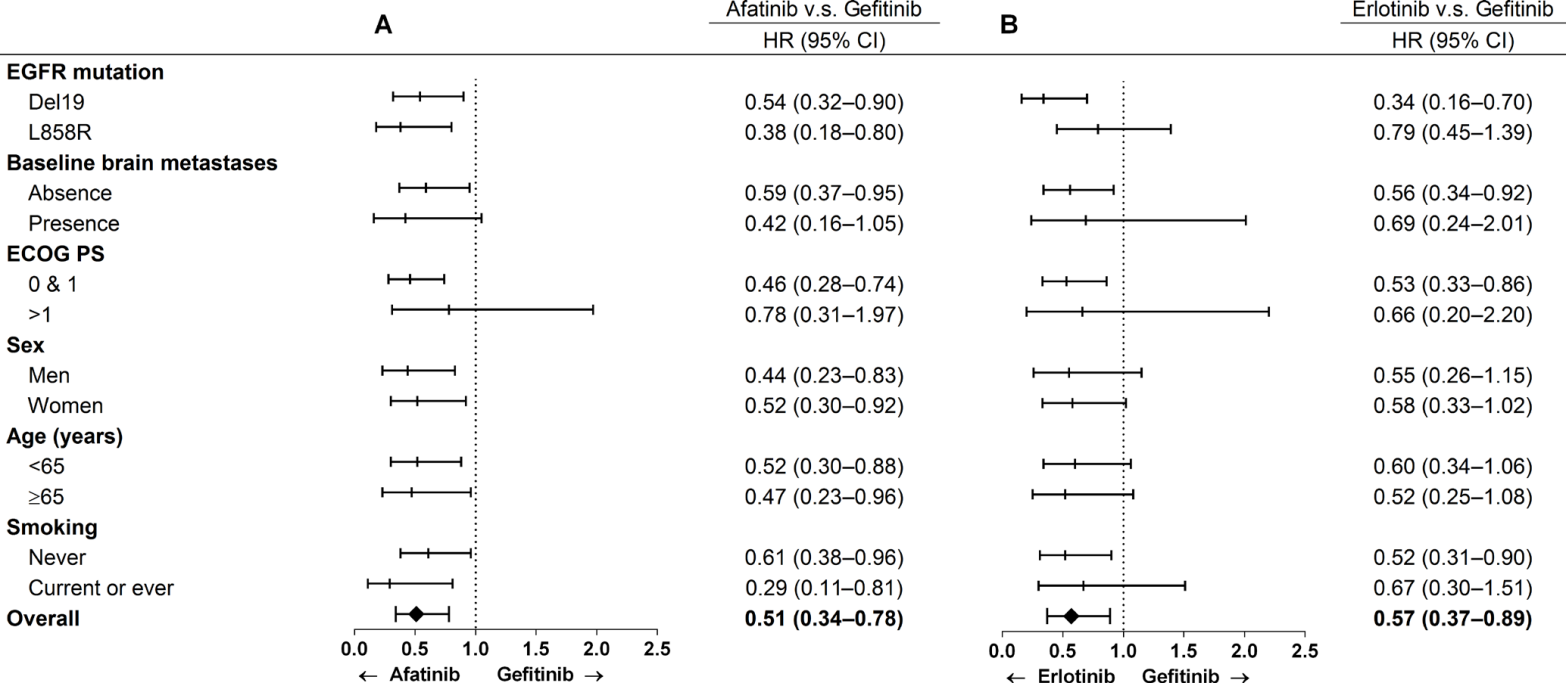
Multivariable analysis of progression-free survival in patients received afatinib v.s gefitinib and erlotinib v.s. gefitinib

**Figure 5:**
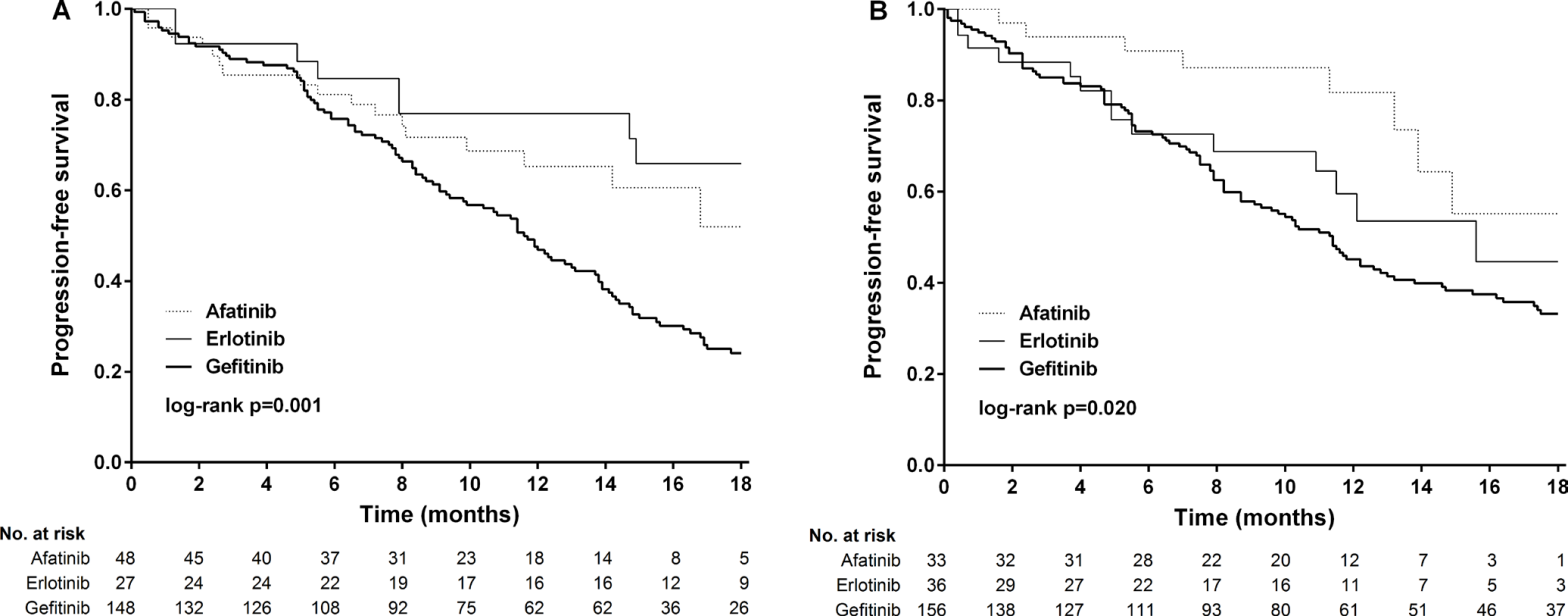
Kaplan-Meier survival curves of progression-free survival of patients received gefitinib, erlotinib and afatinib in (A) exon 19 deletions and (B) Leu858Arg

Multivariate stratified analysis of progression-free survival is shown in Figure 4 and Supplementary Table S3 and S4. After adjusting for covariates including age, sex, smoking, EGFR mutation, baseline brain metastasis and performance status, afatinib reduced the risk of progression in all subgroups except for performance status > 1 (HR, 0.78; 95% CI, 0.31–1.97) and a trend of a reduction in risk in patients with synchronous brain metastasis (HR, 0.42; 95% CI, 0.16–1.05) compared to the gefitinib group. After adjusting for these covariates, erlotinib reduced the risk of progression in the patients with exon 19 deletions (HR, 0.34; 95% CI, 0.16–0.70), without synchronous brain metastasis (HR, 0.56; 95% CI, 0.34–0.92), with a performance status of 0 and 1 (HR, 0.53; 95% CI, 0.33–0.86), and in never smokers (HR, 0.52; 95% CI, 0.31–0.90). Analysis of progression-free survival according to the type of mutation (exon 19 deletions or Leu858Arg) is shown in Figure 5A and 5B. In the patients with exon 19 deletions, afatinib or erlotinib treatment was associated with significantly longer progression-free survival than gefitinib (*p* = 0.001). However, in the patients with the Leu858Arg mutation, afatinib was associated with significantly longer progression-free survival compared to erlotinib or gefitinib (*p* = 0.02).

## DISCUSSION

The recent LUX-Lung 7 trial reported statistically significant benefits in progression-free survival in patients receiving afatinib compared to gefitinib as first-line treatment in patients with non-small cell lung cancer harboring EGFR-activating mutations (HR, 0.73; 95% CI, 0.57–0.95, *p* = 0.017) [23]. Erlotinib, another first-generation tyrosine kinase inhibitor, is not included in this head-to-head trial, although previous studies have indicated a similar efficacy with gefitinib [24–26]. To the best of our knowledge, the current study is the first to investigate differences in progression-free survival between these three EGFR tyrosine kinase inhibitors. Our findings may provide important information for physicians when choosing the first-line treatment for these patients. Consistent with the results of the LUX-Lung 7 trial, afatinib was superior to gefitinib in our study (*p* < 0.001). In addition, erlotinib was superior to gefitinib (*p* = 0.005). In the multivariable comparisons of afatinib and gefitinib, the benefits in progression-free survival were statistically significant and consistent in different subgroup analyses after adjusting for other covariates (HR, 0.51; 95% CI, 0.34–0.78), except for patients with baseline brain metastasis (absence vs. presence) and worse performance status (0 and 1 vs. > 1) indicating the superiority of afatinib, consistent with the findings of the LUX-Lung 7 trial.

Similarly, erlotinib outperformed gefitinib but to a lesser extent (HR, 0.57; 95% CI, 0.37–0.89) in multivariable analysis. With regards to the type of mutation and progression-free survival, afatinib and erlotinib performed better than gefitinib in the patients with exon 19 deletion mutations (*p* = 0.001), and afatinib performed better than erlotinib and gefitinib in the patients with Leu858Arg mutations (*p* = 0.02). This may be because afatinib has a broader inhibitory profile, which can delay resistance mechanisms in both exon 19 deletion and Leu858Arg mutations [6, 27–31]. De novo Thr790Met mutations may also account for this poor response to reversible tyrosine kinase inhibitors [7–9, 32]. However, the incidence of de novo Thr790Met has been reported to range from 0% to 78.9% depending on the molecular testing technique [10, 32–39]. Although the relationship between baseline Thr790Met and exon 19 deletion or Leu858Arg has yet to be elucidated, a higher baseline incidence of Thr790Met mutations has been associated with the Leu858Arg (Table 2). In this study, the baseline incidence of Thr790Met in the patients with Leu858Arg (7/260 = 2.7%) was higher than that in the patients with exon 19 deletions (4/257 = 1.5%). Previous literatures showed only modest activity of second-generation TKIs against Thr790Met in TKI-pretreated or -naïve patients [29, 40, 41]. Nevertheless, in the LUX-Lung 7 trial, afatinib was shown to have a favorable response and to be able to overcome primary resistance in those with Leu858Arg compared to gefitinib. Other head-to-head studies such as ARCHER1050 (ClinicalTrials.gov identifier: NCT01774721) and FLAURA (ClinicalTrials.gov identifier: NCT02296125) trials are currently ongoing, and should provide more insight into these issues.

**Table 2:** Literatures regarding baseline Thr790Met in EGFR-activating mutations

Author	T790M+del19/del 19	T790M+L858R/L858R	Country
Fujita 2012 ^10^	16/22 (72.7%)	12/13 (92.3%)	Japan
Yu 2014 ^32^	4/20 (20.0%)	16/20 (80.0%)	USA
Costa 2014 ^10^	50/84 (59.5%)	29/39 (74.4%)	Spain
Rosell 2011 ^10^	42/78 (53.8%)	36/53 (67.9%)	USA
Li 2014 ^36^	5/28 (17.9%)	10/26 (38.5%)	China
Stahel 2015 ^38^	23/70 (32.8%)	14/39 (35.9%)	Spain
Su 2012 ^10^	4/28 (14.3%)	24/67 (35.8%)	Taiwan
Maheswaran 2008 ^10^	5/16 (31.3%)	2/7 (28.5%)	USA
Lee 2015 ^10^	11/76 (14.5%)	13/48 (27.1%)	South Korea
Hashida 2014 ^10^	5/26 (19.2%)	6/28 (21.4%)	Japan
Sequist 2008 ^40^	0/18 (0%)	2/11 (18.2%)	USA
Ren 2012 ^10^	1/26 (3.8%)	5/42 (11.9%)	China
Janne 2014 ^10^	1/26 (3.8%)	2/23 (8.7%)	Hong Kong, Japan, South Korea, Taiwan, USA
Arrieta 2015 ^10^	3/110 (2.7%)	4/50 (8.0%)	Mexico
Ragazzi 2015 ^10^	3/38 (7.9%)	1/47 (2.1%)	Italy
Inukai 2006 ^10^	1/44 (2.3%)	3/43 (7.0%)	Japan
He 2013 ^10^	7/108 (6.5%)	4/104 (3.8%)	China
Kris 2013 ^35^	3/106 (2.8%)	4/68 (5.9%)	USA
Yang 2013 ^10^	3/237 (1.3%)	6/183 (3.3%)	Asia, Europe, North/South America, Australia, China, Thailand, South Korea
Fukuoka 2011 ^10^	4/136 (2.9%)	3/107 (2.8%)	East Asia
Guo 2015 ^10^	1/104 (0.9%)	2/73 (2.7%)	China
Keam 2014 ^10^	3/180 (1.7%)	2/109 (1.8%)	South Korea
Inoue 2016 ^33^	4/823 (0.5%)	5/681 (0.7%)	Japan
Baek 2015 ^10^	1/287 (0.3%)	1/206 (0.5%)	South Korea
Wu 2011 ^39^	0/258 (0%)	0/260 (0%)	Taiwan

There are several limitations to this study. First, the median follow-up time was less than 1 year in the erlotinib and afatinib arms, and neither arm reached their median progression-free survival. However, the median follow-up time in the gefitinib arm was 12.1 months, which may serve as a good reference compared with previous studies [12–15, 33, 37, 42–44], and 79.3% of these patients had clinical progression or death then. The differences between erlotinib and afatinib compared to gefitinib were evident, although they may be relatively modest with longer follow-up. Nevertheless, this is unlikely to substantively change our results. Second, the number of cases differed in the three arms, and more patients received gefitinib than erlotinib or afatinib. However, there were no significant differences in demographic data except for more patients with a poor performance status in the gefitinib group. In addition, some clinical factors such as pleural effusion were not documented, which may have been a source of confounding. On the other hand, baseline brain metastasis and smoking status were relatively consistent in all treatment arms. These factors have been reported to be prognostic factors in these patients, and thus we adjusted for other covariates to make appropriate comparisons [44, 45]. Third, the BCL2-Like 11 (BIM) deletion polymorphism has been reported to occur in 12.8% to 18.6% of Asians, and to be associated with an inferior response to tyrosine kinase inhibitors [46–48]. Although Lee et al. reported no predictive role of BIM regarding the outcomes of tyrosine kinase inhibitor therapy [49], we did not check this in our study population. Finally, we did not report the side effects in the treatment groups. It is known that afatinib is associated with a higher frequency of diarrhea and rash, and that gefitinib is associated with a higher frequency of liver function abnormalities and interstitial pneumonitis [23, 50]. Our results of progression-free survival are based on real world practice with acceptable dosages of medication.

## MATERIALS AND METHODS

### Study design and participants

This study was conducted at Linkou Chang-Gung Memorial Hospital (LK-CGMH), a university-affiliated medical center with more than 8,00 newly-documented cases of lung cancer a year. In Taiwan, gefitinib has been reimbursed by the National Health Insurance program (NHIP) for the first-line treatment of patients with stage IIIb or IV non-small cell lung cancer with EGFR-activating mutations since June 2011, with erlotinib and afatinib being added in November 2013 and May 2014, respectively. Patients were included into this study if they had: (1) initial or recurrent stage IIIb or IV lung adenocarcinoma that had been diagnosed at LK-CGMH between February 16, 2011 and October 30, 2015; (2) activating somatic EGFR mutations; and (3) treatment with first-line gefitinib, erlotinib, or afatinib. The Institutional Research Ethics Committee of CGMH approved this study.

### Procedures

Lung cancer was pathologically confirmed by a bronchoscopic or CT-guided biopsy, pleural effusion cytology and/or surgical procedures. EGFR mutation analysis was performed in patients with adenocarcinoma, large cell carcinoma, or carcinoma with an adenocarcinoma component such as adenosquamous carcinoma. The mutation analysis was performed by direct sequencing with polymerase chian reaction or with SCORPION technology in combination with an Amplified Refractory Mutation System (ARMS, QIAGEN, Hilden, Germany) or competitive allele-specific TaqMan PCR (Cast-PCR, Applied Biosystems, Foster City, CA) with genomic DNA from paraffin-embedded tissue (Supplement Table S1). [51, 52].

Patients in the gefitinib group received 250 mg orally once daily, with a reduction in the dose being permitted on an individual basis. The patients in the erlotinib group received 150 mg orally once daily, and the dose could be reduced to 100 mg if there were intolerable side effects. Similarly, the patients in the afatinib group received 40 mg orally once daily, with a reduction to 30 mg being permitted if necessary. Chest computed tomography or other clinical imaging modalities (chest radiography, brain magnetic resonance imaging, bone scan, or positron emission tomography-computed tomography) were arranged every 3 months to re-evaluate the disease status, and if the disease was considered to be under control (either a complete response, partial response or stable disease according to RECIST 1.1) [53], the individual TKI would be prescribed and reimbursed again by NHIP after re-application.

### Outcomes and statistical analysis

Progression-free survival was calculated from the time of initiating tyrosine kinase inhibitor treatment to the time of clinical progression or death, whichever occurred first. The time of “clinical progression” was defined as the date that radiographic imaging was judged by both the physician and radiologist to be clinically significant, and warranting a change in therapy. Kaplan-Meier curves were used to estimate survival, and the log-rank test was used to compare times to events between groups. Multiple analyses and stratified analyses were performed using Cox proportional hazards regression models (hazard ratio, HR). All reported *p* values were two-sided, and adjustments were made for multiple comparisons. All analyses were performed using SAS version 9.4 (SAS Inc., Cary, NC).

### Role of the funding source

The authors declare no conflicts of interest and no funding source.

## CONCLUSIONS

Afatinib and erlotinib had a significantly longer progression-free survival than gefitinib in the first-line treatment of patients with lung adenocarcinoma harboring common EGFR-activating mutations. The patients with Leu858Arg mutations who received afatinib had a longer progression-free survival than those receiving gefitinib or erlotinib, which may have been due to a higher baseline incidence of Thr790Met. Further clinical trials are warranted to validate these findings.

## SUPPLEMENTARY MATERIALS


