# Supplementary material for: Analysis of progression-free survival of first-line tyrosine kinase inhibitors in patients with non-small cell lung cancer harboring leu858Arg or exon 19 deletions
Source: Oncotarget. 2016 Dec 7;8(1):1343–53. doi: 10.18632/oncotarget.13815 (PMC5352060; doi:10.18632/oncotarget.13815)
Supplement: Supplementary file 1 [file oncotarget-08-1343-s001.pdf]

Analysis of progression-free survival of first-line tyrosine kinase inhibitors in patients with non-small cell lung cancer harboring leu858Arg or exon 19 deletions

Supplementary Materials

Supplementary Table S1: Methods of EGFR mutation analysis among first-line TKI users

	EGFR-TKIs					
	Gefitinib		Erlotinib		Afatinib	
	n	(%)	n	(%)	n	(%)
Total	304		63		81	
Testing method						
Competitive allele-specifit TaqMan PCR	48	(15.8%)	10	(15.9%)	9	(11.1%)
PCR/direct sequencing	149	(49.0%)	24	(38.1%)	18	(22.2%)
Scorpions & ARMS	95	(31.3%)	29	(46.0%)	53	(65.4%)
Nil*	12	(3.9%)	0	(0%)	1	(1.2%)

*patient receive EGFR mutation analysis in another hospital.

Supplementary Table S2: Distribution of EGFR compound mutations among first-line TKI users

EGFR mutations		EGFR-TKIs		
		Gefitinib	Erlotinib	Afatinib
Exon 18	Substitution	4<br>(E709G, E709K, E709V, Q701L)	0	0
	Polymorphism	1<br>(2421 G/A polymorphism)	0	0
Exon 19	Insertions	12	2	3
	Substitution	1<br>(G729A)	1<br>(G747S)	0
	Polymorphism	1<br>(2469 C/T polymorphism)	0	0
Exon 20	Substitution	5<br>(R776H, R776C, S768I, T790M, T790M)	3<br>(E804K, T790M, T790M)	2<br>(T790M, T790M)
	Polymorphism	44<br>(2703 G/A polymorphism x2, 2607 G/A polymorphism x42)	6<br>(2361 G/A polymorphism x1, 2607 G/A polymorphism x5)	8<br>(2361 G/A polymorphism x3, 2364 C/T polymorphism x1, 2607 G/A polymorphism x4)
Exon 21	Substitution	2<br>(L833V, V834L)	0	0

Supplementary Table S3: Multivariable analysis of progression-free survival in patients received afatinib or gefitinib

Subgroup	Gefitinib (n)		Afatinib (n)			
	Events	Patients	Events	Patients	HR (95% CI)[a]	$P_{interaction}$
EGFR mutation						0.443
Del19	102	148	17	48	0.54 (0.32–0.90)	
L858R	97	156	8	33	0.38 (0.18–0.80)	
Baseline brain metastases						0.589
Absence	157	244	20	64	0.59 (0.37–0.95)	
Presence	42	60	5	17	0.42 (0.16–1.05)	
ECOG PS						0.300
0 & 1	147	231	19	70	0.46 (0.28–0.74)	
> 1	52	73	6	11	0.78 (0.31–1.97)	
Sex						0.580
Men	74	114	11	39	0.44 (0.23–0.83)	
Women	125	190	14	42	0.52 (0.30–0.92)	
Age (years)						0.856
< 65	109	154	16	52	0.52 (0.30–0.88)	
≥ 65	90	150	9	29	0.47 (0.23–0.96)	
Smoking						0.172
Never	143	226	21	63	0.61 (0.38–0.96)	
Current or ever	56	78	4	18	0.29 (0.11–0.81)	
Overall	199	304	25	81	0.51 (0.34–0.78)	

Abbreviations: ECOG PS, Eastern Cooperative Oncology Group performance status; HR, hazard ratio.

[a]Multivariable analysis is by Cox proportional hazards model. Adjusted for covariate factors, including age, sex, smoking, EGFR mutation, baseline brain metastases, and ECOG PS.

Supplementary Table S4: Multivariable analysis of progression-free survival in patients received erlotinib or gefitinib

Subgroup	Gefitinib (n)		Erlotinib (n)		HR (95% CI)[a]	$P_{interaction}$
	Events	Patients	Events	Patients		
EGFR mutation						0.096
Del19	102	148	8	27	0.34 (0.16–0.70)	
L858R	97	156	14	36	0.79 (0.45–1.39)	
Baseline brain metastases						0.504
Absence	157	244	18	52	0.56 (0.34–0.92)	
Presence	42	60	4	11	0.69 (0.24–2.01)	
ECOG PS						0.473
0 & 1	147	231	19	56	0.53 (0.33–0.86)	
>1	52	73	3	7	0.66 (0.20–2.20)	
Sex						0.778
Men	74	114	8	24	0.55 (0.26–1.15)	
Women	125	190	14	39	0.58 (0.33–1.02)	
Age (years)						0.802
<65	109	154	14	34	0.60 (0.34–1.06)	
≥65	90	150	8	29	0.52 (0.25–1.08)	
Smoking						0.492
Never	143	226	15	48	0.52 (0.31–0.90)	
Current or ever	56	78	7	15	0.67 (0.30–1.51)	
Overall	199	304	22	63	0.57 (0.37–0.89)	

Abbreviations: ECOG PS, Eastern Cooperative Oncology Group performance status; HR, hazard ratio.
[a]Multivariable analysis is by Cox proportional hazards model. Adjusted for covariate factors, including age, sex, smoking, EGFR mutation, baseline brain metastases, and ECOG PS.
